# *MFRP* variations cause nanophthalmos in five Chinese families with distinct phenotypic diversity

**DOI:** 10.3389/fgene.2024.1407361

**Published:** 2024-07-15

**Authors:** Zhen Li, Runqing Ma, Meijiao Ma, Xue Xiao, Xiaolong Qi, Hongjuan Ma, Xunlun Sheng, Weining Rong

**Affiliations:** ^1^ Ningxia Eye Hospital, People’s Hospital of Ningxia Hui Autonomous Region, Ningxia Medical University, Yinchuan, China; ^2^ Gansu Aier Optometry Hospital, Lanzhou City, China

**Keywords:** nanophthalmos, whole exome sequencing, genetic variant, clinical, MFRP

## Abstract

**Purpose:**

Nanophthalmos is a congenital ocular structural anomaly that can cause significant visual loss in children. The early diagnosis and then taking appropriate clinical and surgical treatment remains a challenge for many ophthalmologists because of genetic and phenotypic heterogeneity. The objective of this study is to identify the genetic cause of nanophthalmos in the affected families and analyze the clinical phenotype of nanophthalmos with *MFRP* gene variation (Microphthalmia, isolated; OMIM#611040 and Nanophthalmos 2; OMIM#609549, respectively).

**Methods:**

Comprehensive ophthalmic examinations were performed on participants to confirm the phenotype. The genotype was identified using whole exome sequencing, and further verified the results among other family members by Sanger sequencing. The normal protein structure was constructed using Alphafold. Mutant proteins were visualized using pymol software. Pathogenicity of identified variant was determined by *in silico* analysis and the guidelines of American College of Medical Genetics and Genomics (ACMG). The relationship between genetic variants and clinical features was analyzed.

**Results:**

Five nanophthalmos families were autosomal recessive, of which four families carried homozygous variants and one family had compound heterozygous variants in the *MFRP* gene. Both family one and family three carried the homozygous missense variant c.1486G>A (p.Glu496Lys) in the *MFRP* gene (Clinvar:SCV005060845), which is a novel variant and evaluated as likely pathogenic according to the ACMG guidelines and *in silico* analysis. The proband of family one presented papilloedema in both eyes, irregular borders, thickened retinas at the posterior pole, tortuous and dilated retinal vessels, and indistinguishable arteries and veins, while the proband of family three presented uveal effusion syndrome-like changes in the right eye. In families one and 3, despite carrying the same gene variant, the probands had completely different clinical phenotypes. The homozygous nonsense variant c.271C>T (p.Gln91Ter) (Clinvar:SCV005060846) of the *MFRP* gene was detected in family 2, presenting shallow anterior chamber in both eyes, pigmentation of peripheral retina 360° from the equator to the serrated rim showing a clear demarcation from the normal retina in the form of strips. Family four proband carried the homozygous missense variant c.1411G>A (p.Val471Met) in the *MFRP* gene (Clinvar:SCV005060847), family five proband carried compound heterozygous missense variants c.1486G>A (p.Glu496Lys) and c.602G>T (p.Arg201Leu) in the *MFRP* gene (Clinvar:SCV005060848), which is a novel variant and evaluated as likely pathogenic according to the ACMG guidelines and *in silico* analysis, and they all presented clinically with binocular angle-closure glaucoma, family four also had retinal vein occlusion in the right eye during the follow-up.

**Conclusion:**

In this study, pathogenic variants of the *MFRP* gene were detected in five nanophthalmos families, including two novel variants. It also revealed a distinct phenotypic diversity among five probands harboring variants in the *MFRP* gene. Our findings extend the phenotype associated with *MFRP* variants and is helpful for ophthalmologists in early diagnosis and making effective treatment and rehabilitation strategies.

## 1 Introduction

Microphthalmia (MCO) is a genetically heterogeneous ocular congenital structural anomaly that can cause significant visual loss in children with an incidence of 2–17/100,000, and 9/100,000 in the Chinese population (Hu et al., 2011; Carricondo et al., 2018; Ally et al., 2022). In clinical practice, MCO can be subdivided into complete microphthalmia (nanophthalmos) and posterior microphthalmia (posterior nanophthalmos) according to whether the anterior segment is involved or not (Saifuddin Adeel et al., 2023). Both types can be associated with retinitis pigmentosa, macular retinoschisis and optic disc drusen. Complete microphthalmos, also known as nanophthalmos, is the most common type in the clinic, which is defined as a small eye with microcornea (axial length <20 mm and corneal diameter <10 mm at adults)accompanied by other ocular disorders. In addition to the overall shortening of ocular axial length, it may be accompanied by such features as abnormal scleral thickening and a shallow anterior chamber, which makes it highly susceptible to angle-closure glaucoma, exudative retinal detachment and choroidal detachment.

Nanophthalmos is the result of arrested eyeball development during early embryogenesis, and thus has a strong genetic basis, featuring a variety of inheritance patterns inclusive of autosomal dominant (AD), autosomal recessive (AR) and X-linked recessive (XL). To date, the following six genes, *PRSS56, MFRP, TMEM98, CRB1, BEST1* and *MYRF*, have been reported to be associated with the development of nanophthalmos (Garnai et al., 2019; Almoallem et al., 2020; Prasov et al., 2020). Notably, patients carrying biallelic mutations in the *PRSS56* or *MFRP* genes have shorter axis oculi and more severe clinical phenotypes than patients carrying genetic mutations in the *TMEM9*8 or *MYRF* genes (Siggs et al., 2020).

So far, 116 variants of *MFRP* gene leading to nanophthalmos have been reported, of which 47 variants are pathogenic, 45 variants are benign, and 24 variants have uncertain significance (https://databases.lovd.nl/shared/genes/). Wasmann et al. (Wasmann et al., 2014) had reported 14 different described *MFRP* mutations: two of them were single amino acid substitutions at extremely conserved sites and 12 caused severe truncation of the protein. All of these cases presented with high hyperopia, but the effect of the mutation on retinal rod photoreceptor function was different between individuals, and the clinical spectrum of age of onset and severity of disease was quite variable. Understanding the genetic mechanisms of nanophthalmos pathogenesis will ultimately help us provide potential markers for genetic diagnosis and the development of innovative therapies for this disease.

In this paper, we describe five patients with nanophthalmos carrying variations in *MFRP* gene from five Chinese families and discuss the phenotypic characteristics of individuals with different genotypes, providing reliable molecular diagnosis for nanophthalmos and offering options for Eugenics for these kinds of family members.

## 2 Materials and methods

### 2.1 Ethical approval

The Ningxia Hui Autonomous Region People’s Hospital Ethics Committee granted approval for the study (approval number 2022-KJCG-006), and everyone or legal guardian who took part in it signed an informed consent form and strictly followed the Declaration of Helsinki.

### 2.2 Diagnostic criteria

Referring to Ghose et al. (Ghose et al., 1985) who used ultrasonographically measured ocular axial length of less than 2 standard deviations from the mean value of normal subjects as a criterion, the patients with nanophthalmos whose axial length was less than 20 mm and who had no previous history of ocular surgery and were not accompanied by eyeballs and other systemic deformities were included in this study.

### 2.3 Clinical data collection

Five nanophthalmos pedigrees were recruited from the Ningxia Eye Hospital, People’s Hospital of Ningxia Hui Autonomous Region from 2020 to 2021. The patients and their major family members were asked about their ocular history, marital and family history. The detailed ophthalmologic examinations were performed, including best-corrected visual acuity (BCVA), slit-lamp microscopy, fundus examination with photographs (Daytona (P200T), OPTOS), optical coherence tomography (OCT, HDOCT4000, Carl Zeiss Meditec, United States), electroretinogram (ERG) and ocular axial measurement (SW-2100,SUOER) and ultrasound biomicroscopy (UBM,SW-3200L,SUOER). ERG was detected and analyzed with reference to the standards of the International Society for Clinical Electrophysiology of Vision (ISCEV).

### 2.4 Whole exome sequencing

Whole exome sequencing was performed on probands and their parents (Trio whole-genome exome sequencing model). Agilent SureSelect exome capture kit was used to perform the whole-genome exome capture, and high-throughput sequencer (Illumina) was used to perform sequencing subject to a depth of 100 ×. The raw sequencing data were processed by Illumina basecalling Software 1.7 and subsequently compared with the NCBI human genomic DNA reference sequence (NCBI build 37.1). Single nucleotide variation (SNV) was analyzed by SOAP software (http://soap. genomics. org.cn) while insert-deletion (Indel) was analyzed by BWA software (bio-bwa.sourceforge.net/) to obtain all the variants occurring in the DNA sequences in the samples. Common variants (which are usually considered that MAF (minor allele frequency) > 1%) were filtered from the database (db135). The variants that do not affect the structure and function of the protein were then filtered out. After stepwise filtering, the number of variants shared by all patients in the families was screened out, and then variants unavailable with the diseased relatives in the families were filtered to obtain the candidate pathogenic gene variants. Sanger validation was used to exclude false positives for candidate pathogenic genetic variants, and co-segregation was further verified in the normal family members.

### 2.5 Pathogenicity analysis of genetic variants

The American College of Medical Genetics and Genomics (ACMG) established Standards and Guidelines for Interpretation of Sequence Variants in 2015, which were used to evaluate the pathogenicity of novel variations for genetic variation. MAF<0.005 was used as the criteria to exclude benign variants by reference to the databases for East Asian populations Allele frequencies available with 1000 Genomes Project (1000G, http://browser.1000genomes.org) and Exome Aggregation Consortium (http://exac.broadinstitute.org/). Polyphen2 (http://genetics.bwh.harvard. edu/pph2), SIFT (http://sift.jcvi.org),REVEL (https://www.ncbi.nlm.nih.gov/pmc/articles/PMC5065685/), CADD (https://cadd.gs.washington.edu/score) and Mutation Taster (http://mutationtaster.org/) were used for pathogenicity prediction. Measurements of the conservation of gene sequences across species in evolution have been made using websites like GERP++ (https://bio.tools/gerp). Variants were classified as uncertain clinical significance when at least one of four predictions had a benign outcome or when there was insufficient evidence of pathogenicity. When all predictions turned out to be accurate, variations were categorized as potentially pathogenic when used in conjunction with further data. Pathogenic variants were defined as frameshift, nonsense, and variants with experimental proof of causing loss of protein function. For the conservativeness study of variant loci, the online analysis tool Multalin (http://sacs.ucsf.edu/cgi-bin/multalin.py) was employed. Alphafold was used to construct the normal protein structure, and pymol software was used to make visualized analysis of the mutant protein.

## 3 Results

### 3.1 Pedigree 1

**FIGURE 1:**
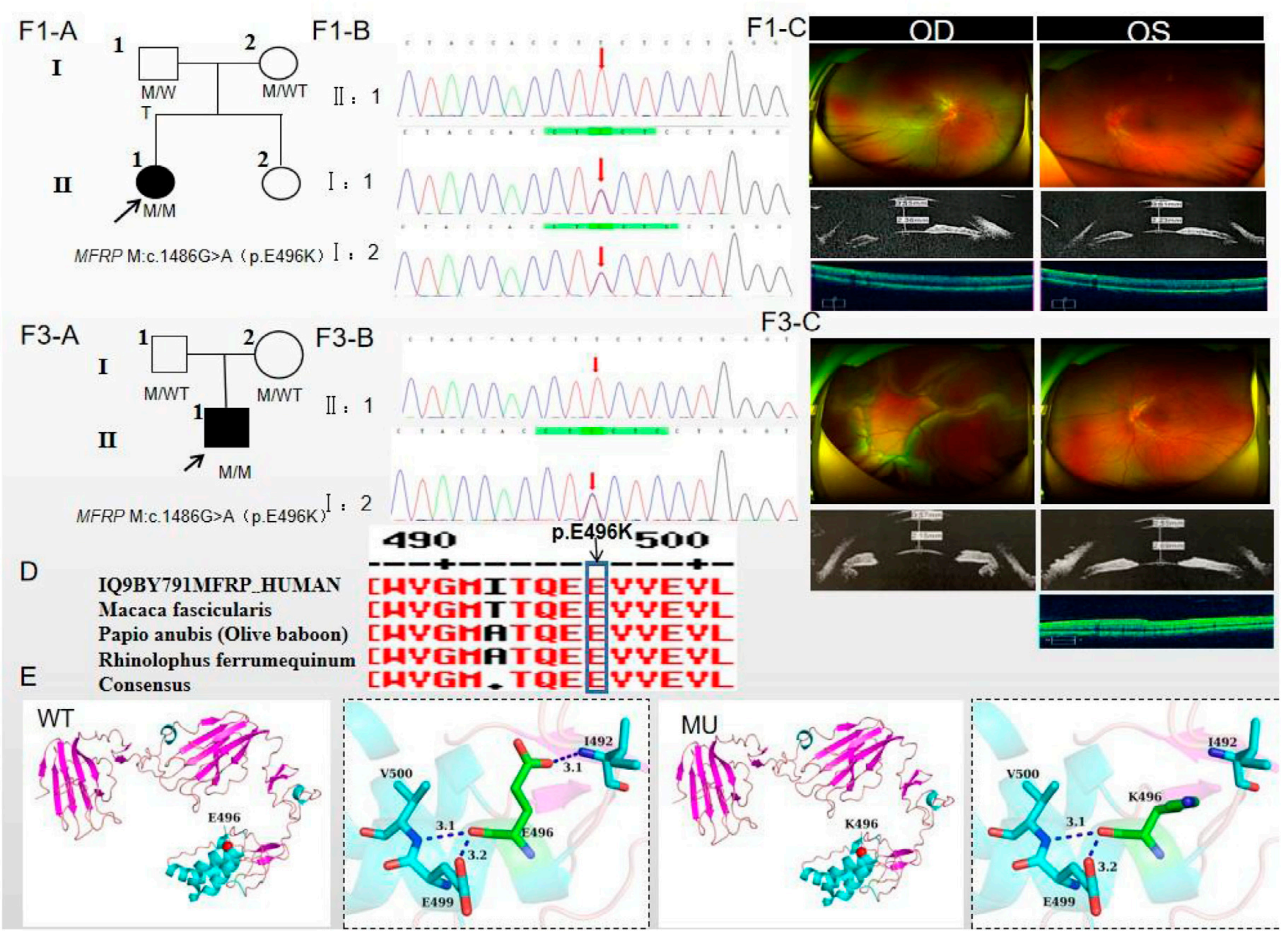
Sequence analysis and clinical examination of Family one and Family 3. **(F1-A)** Pedigree of Family 1. The filled black symbols represent the affected members, and the arrow denotes the proband. **(F1-B)** Sequence chromatograms of identified mutations of Family 1. **(F1-C)** The fundus of both eyes of Family 1: The fundus examination revealed papilloedema in both eyes, no reflection in the macular fovea, thickened retinas at the posterior pole, tortuous and dilated retinal vessels, and indistinguishable arteries and veins. UBM showed the anterior chamber depth of right eye and left eye were 2.36 mm and 2.23 mm, respectively. OCT showed the macular mildly edematous, and the fovea centralis disappeared in both eyes. **(F3-A)** Pedigree of Family 3. The filled black symbols represent the affected members, and the arrow denotes the proband. **(F3-B)** Sequence chromatograms of identified mutations of Family 3. **(F3-C)** The fundus of both eyes of Family 3: The fundus examination revealed that only the posterior retina in the right eye was in normal position as flat and attached, while the detached retina appeared to have a ring-shaped shape and was smooth and free of hiatus; In the left eye the optic disc was small and congested, with clear borders, and the retinal vessels were dilated, with indistinct arteries and veins. UBM showed the anterior chamber depth of right eye and left eye were 2.15 mm and 2.69 mm, respectively. OCT showed a wrinkled, elevated macular area and the fovea centralis disappeared of the left eye. Macular OCT could not be performed in the right eye due to retinal detachment. **(D)** The homology of amino acid sequences between human *MFRP* and other species. The amino acid at position 496 is highly conserved among species. The mutated residue 496 is boxed and indicated. **(E)** The protein structure suggested that p. Glu496Lys mutation resulted in the substitution of the polar negatively charged glutamic acid by the polar positively charged lysine at site 496 of the MFRP protein.

The proband of family 1, a female, 7 years old, complained of reduced vision in both eyes for 1 year. Her parents denied family history and consanguineous marriage history (Figure F1-A). The BCVA was 0.05 in both eyes. The axial length of right eye and left eye were 15.15 mm and 14.71 mm, respectively. The anterior chamber depth of right eye and left eye were 2.36 mm and 2.23 mm, respectively (Figure F1-C). The fundus examination revealed pseudo-papilloedema in both eyes, no reflection in the macular fovea, thickened retinas at the posterior pole, tortuous and dilated retinal vessels, and indistinguishable arteries and veins (Table 1; Figure F1-C). OCT showed the macular mildly edematous, and the fovea centralis disappeared in both eyes (Figure F1-C). Binocular color ultrasonography suggested that the eye axes of both eyes were short and the eyeball walls were thickened. ERG findings suggested that the scotopic vision 0.01 and responses of flicker were in the normal range, whereas the b-wave amplitudes in the photopic 3.0 and scotopic 3.0 responses were significantly higher, and that the function of the amacrine cells in both eyes was basically normal (Table 2). As confirmed by whole exome sequencing and Sanger sequencing, the proband carried a homozygous missense variant c.1486G>A (p.Glu496Lys) in the *MFRP* gene, and both the phenotypically normal father and mother carried the same heterozygous variant, suggesting co-segregation of genotypes and clinical phenotypes (PP1_Supporting) (Figure F1-B; Table 3), which is consistent with an autosomal recessive pattern of inheritance. The variant has not been detected in the East Asian population database (ExAC_ EAS), gnomAD database and it has not been reported before (PM2_Moderate). A variety of bioinformatics computing software indicated the deleterious impact of this variant (PP3_Supporting). Moreover, proteomic conservation analysis suggested that the amino acid at site 496 was highly conserved across multiple species (Figure 1D), indicating that variants at such site are likely to have an impact on the structure and function of the MFRP protein. In the wild-type MFRP protein, site 496 was a polar negatively charged glutamic acid, and its amino acid backbone formed a hydrogen bond with the O atom of the uncharged polar valine at site 500 and the negatively charged glutamic acid at site 499, and such mutation resulted in the substitution of the polar negatively charged glutamic acid by the polar positively charged lysine at site 496 of the MFRP protein. These alterations in amino acid interactions did not lead to alterations in backbone interactions, while the mutation in the structure of the side chain led to alterations in interactions with 1,492, which resulted in alterations in protein structure and function (Figure 1E). According to the ACMG guidelines, the c.1486G>A (p.Glu496Lys) missense variant was rated as PP4 due to the phenotype of the missense variant carrier was highly consistent with nanophthalmos, and genetic testing report from credible sources suggested that the variant was pathogenic as PP5. Therefore, the c.1486G>A (p.Glu496Lys) missense variant was classified as a likely pathogenic (PP1+PM2+PP3+PP4+PP5).

**TABLE 1:** Clinical findings in 5 Chinese families with Nanophthal

Patient	Male	Age (y)	Refraction		BCVA		AL,mm		IOP,mmHg		ACD,mm		Fundus examination	
			OD	OS	OD	OS	OD	OS	OD	OS	OD	OS	OD	OS
F1	female	7	+14.00DS/+1.00DC*80°	+15.50DS/+1.00DC*105°	0.05	0.05	15.15	14.71	14.1	16	2.36	2.23	Papilloedema, dilated retinal vessels	Papilloedema, dilatd retinal vessels
F2	female	17	+13.25DS/+1.75DC*75°	+13.25DS/+1.75DC*105°	0.1	0.15	15.57	15.59	15.5	18	2.26	2.23	pigmentation of peripheral retina	pigmentation of peripheral retina
F3	male	19	+16.50DS	+14.00DS	0.02	0.12	16.02	16.68	16	19	2.15	2.69	Peripheral retinal detachment	optic disc was small, retinal vessels were dilated
F4	female	41	+8.75DS/+1.50DC*10°	+8.75DS/+1.50DC*165°	0.04	0.15	18.25	18.30	27	23	2.00	2.17	C/D ratio was 0.8, with markedly dilated retinal veins, Retinal hemorrhage	C/D ratio was 0.8, with mildly dilated retinal veins
F5	female	43	(+12.50DS/+1.50DC*20°	+11.75DS/+2.00DC*150°	0.15	0.2	15.28	15.03	30	28	1.91	2.00	C/D ratio was 0.8, with mildly dilated retinal veins	C/D ratio was 0.8, with mildly dilated retinal veins

OD, right eye; OS, left eye;BCVA, Best Corrected Visual Acuity;AL, Axial length;ACD, anterior chamber depth.

**TABLE 2:** Eletroretinogram analysis of Chinese families with Nanophthal

	Scotopic 0.01 ERG		Scotopic 3.0 ERG				Scotopic 3.0 oscillatory potentials		Photopic 3.0 ERG				Photopic 3.0 flicker	
	b-wave (μV)		a-wave (μV)		b-wave (μV)		OPs(μV)		a-wave (μV)		b-wave (μV)		N1-P1 (μV)	
	OD	OS	OD	OS	OD	OS	OD	OS	OD	OS	OD	OS	OD	OS
Family1	101	113	194	227	472	564	113	113	49.1	26.2	146	176	95.8	145
Family2	12.5	25.5	30.6	35.5	120	152	23.4	35.6	21.8	22.8	86.3	98.2	35.3	44.7
Family3	31.1	83	35.4	98.8	50.9	241	17.9	80.5	12.2	52.1	15.2	97.4	7.47	53
Family4	135	296	353	442	802	913	97.2	180	27.2	51.4	264	250	189	213
Family5	112	99	78	98	412	398	81	72	31	47	88.3	97.2	22.8	42.1
Normal range	97–170		170		320–470		70–114		30–40		87–124		87–130	

**TABLE 3:** Pathogenicity analyses of variants in 5 Chinese families with Nanophthal

	Proband1	Proband2	Proband3	Proband4	Proband5	
Gene	MFRP	MFRP	MFRP	MFRP	MFRP (M1)	MFRP (M2)
Nucleotide	c.1486G>A	c.271C>T	c.1486G>A	c.1411G>A	c.1486G>A	c.602G>T
Amino acid	p.Glu496Lys	p.Gln91Ter	p.Glu496Lys	p.Val471Met	p.Glu496Lys	p.Arg201Leu
NM_	NM_031433.4	NM_031433.4	NM_031433.4	NM_031433.4	NM_031433.4	NM_031433.4
Exon	Exon12	Exon3	Exon12	Exon12	Exon12	Exon5
Inheritance	Father& Mother	Father& Mother	Father& Mother	Father& Mother	Father& Mother	Father& Mother
1000g2015aug_all	-	-	-	0.0002	-	-
1000g2015aug_eas	-	-	-	0.001	-	-
esp6500siv2_all	-	-	-	-		
all_gnomAD	0.000024	0.000004	0.000024	0.000012	0.000024	0.000004
eas_gnomAD	0.000326	0	0.000326	0.000054	0.000326	0
SIFT_	T	-	T	-	T	T
Polyphen2_HDIV_	D	-	D	D	D	D
Polyphen2_HVAR_	P	-	P	D	P	P
LRT	D	N	D	D	D	D
MutationTaster	D	D	D	D	D	D
MutationAssessor	M	-	M	M	M	M
FATHMM_	T	-	T	-	T	T
PROVEAN_	N	-	N	-	N	N
CADD_	3.555	7.977	3.555	3.976	3.555	3.111
CADD_phred score	24.9	40	24.9	27.5	24.9	23.7
GERP++	5.27	4.54	5.27	5.26	5.27	4.15
ACMG Category	likely pathogenic	pathogenic	likely pathogenic	likely pathogenic	likely pathogenic	
ACMG Evidence	PP1+PM2+PP3+PP4+PP5	PVS1+PP1+PM2+PP3	PP1+PM2+PP3+PP4+PP5	PP1+PM2+PP3+PP4+PP5	PM2+ PP1+PP3+PP4+PP5	

### 3.2 Pedigree 2

**FIGURE 2:**
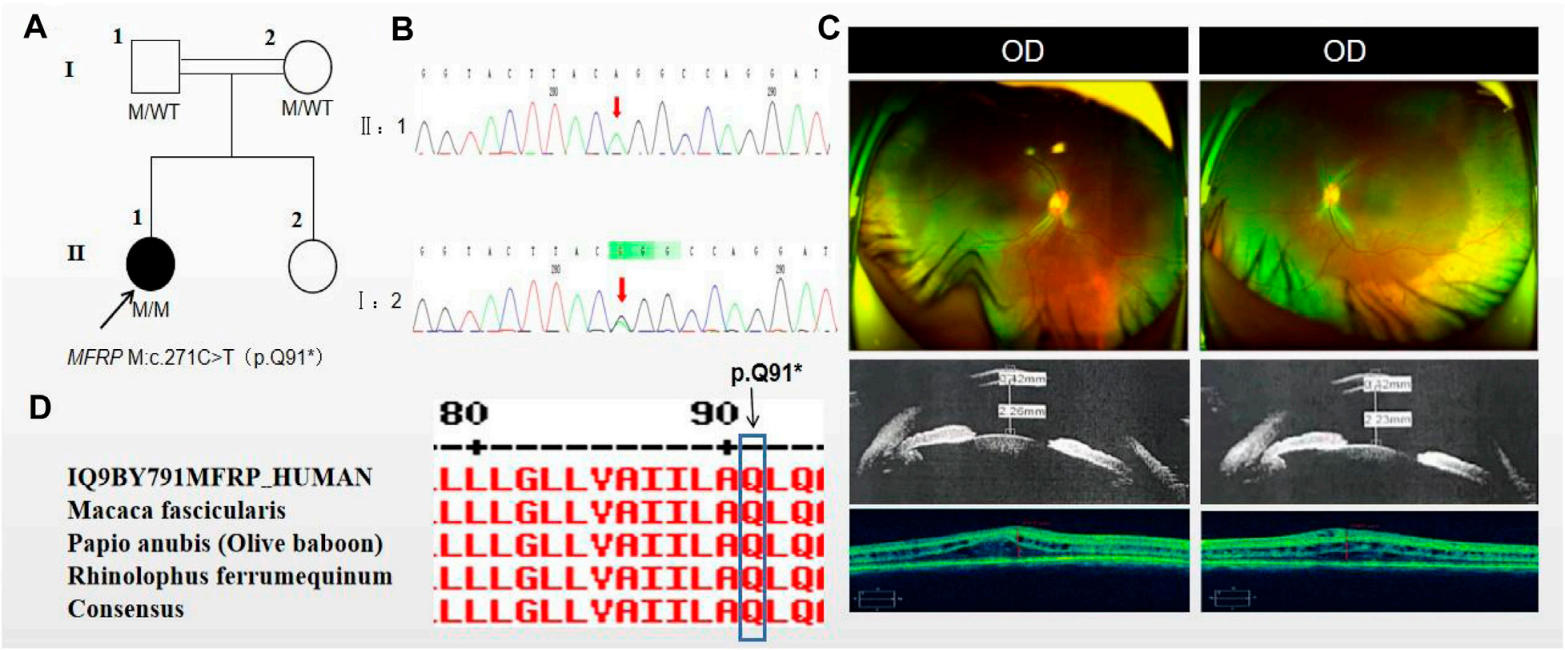
Sequence analysis and clinical examination of Family 2. **(A)** Pedigree of Family 2. The filled black symbols represent the affected members, double horizontal line represent consanguinity and the arrow denotes the proband. **(B)** Sequence chromatograms of identified mutations. **(C)** The fundus of both eyes:The fundus examination revealed slightly smaller optic discs in both eyes with clear borders visible, pigmentation of peripheral retina 360°from the equator to the serrated rim showing a clear demarcation from the normal retina in the form of strips. UBM showed the anterior chamber depth of right eye and left eye were 2.26 mm and 2.23 mm, respectively. OCT showed cystic dilatation of the fovea centralis, separation between retinal neuroepithelial layers, and visible small cystic cavities segmented by columnar connections. **(D)** The homology of amino acid sequences between human *MFRP* and other species. The amino acid at position 91 is highly conserved among species. The mutated residue 91 is boxed and indicated.

**FIGURE 3:**
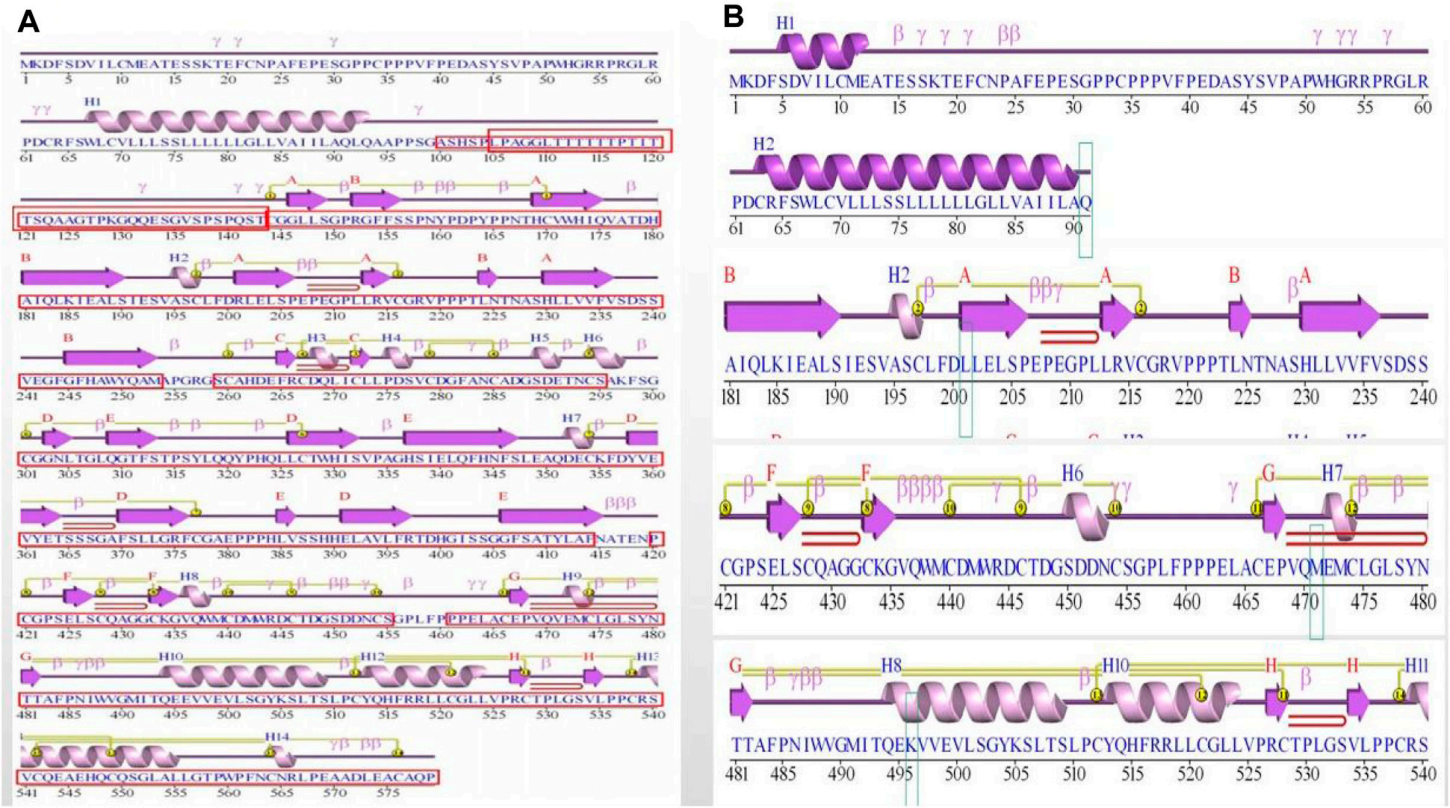
Figure of secondary structure of the protein. **(A)** Protein secondary structure in wild-type (WT). The functional domains were boxed and indicated in red. **(B)** Protein secondary structure in mutation site. The mutated residue 91, 201, 471 and 496 were boxed and indicated.

The proband of family 2, a female, age of 17, complained of progressively reduced vision in both eyes for 10 years accompanied by night blindness for 8 years. Parents are consanguineous marriage (Figure 2A). She had horizontal nystagmus in both eyes. BCVA was 0.1 in the right eye and 0.15 in the left eye. The axial length of right eye and left eye were 15.57 mm and 15.59 mm, respectively. The anterior chamber depth of right eye and left eye were 2.26 mm and 2.23 mm, respectively (Figure 2C). Both eyes demonstrated shallow anterior, slightly smaller optic discs in both eyes with clear borders visible on fundus examination, pigmentation of peripheral retina 360° from the equator to the serrated rim showing a clear demarcation from the normal retina in the form of strips (Table 1; Figure 2C). OCT showed cystic dilatation of the fovea centralis, separation between retinal neuroepithelial layers, and visible small cystic cavities segmented by columnar connections (Figure 2C). ERG findings suggested severe decreases in the b-wave amplitude in the scotopic 0.01 and 3.0 responses, and mild decreases in the b-wave amplitude in the photopic 3.0 response and severe decreases in the flicker response (Table 2). As confirmed by whole exome sequencing and Sanger sequencing, the proband carried a homozygous nonsense variant c.271C>T (p.Gln91Ter)in the *MFRP* gene, and the phenotypically normal father and mother carried the same heterozygous variant, suggesting co-segregation of genotypes and clinical phenotypes (PP1_Supporting) (Figure 2B; Table 3), which was consistent with an autosomal recessive pattern of inheritance. Such variant was not detected or reported in the East Asian population database (ExAC_ EAS) and the gnomAD database (PM2_Moderate). Such mutation caused a change in the glutamine at site 91 to a termination codon, leading to a premature termination of polypeptide chain synthesis (Figure 3). The majority of proteins produced were inactive or lost their usual function (PVS1 - very Strong). Various bioinformatics computing software indicated the deleterious impact of this variant (PP3 - supporting). In addition, proteomic conservation analysis suggested that the amino acid at position 91 was substantially conserved across several taxa (Figure 2D), suggesting that variants at such site were likely to have an impact on the structure and function of the MFRP protein. Therefore, according to the ACMG guidelines, we consider the homozygous nonsense variant c.271C>T (p.Gln91Ter) of *MFRP* as pathogenic (PVS1+PP1+PM2+PP3).

### 3.3 Pedigree 3

The proband of family 3, a male, age of 19, complained of sudden blurring of vision in the right eye for 11 days. He denied a family history of hereditary disease and consanguineous marriage history (Figure 1F3-A). BCVA was 0.02 in the right eye and 0.12 in the left eye. The axial length of right eye and left eye were 16.02 mm and 16.28 mm, respectively (Figure 1F3-C). The anterior chamber depth of right eye and left eye were 2.15 mm and 2.69 mm, respectively. There was no obvious abnormality in the anterior segment of both eyes. Fundus examination suggested that only the posterior retina in the right eye was in normal position as flat and attached, while the detached retina appeared to have a ring-shaped shape and was smooth and free of hiatus; in the left eye the optic disc was small and congested, with clear borders, and the retinal vessels were dilated, with indistinct arteries and veins (Table 1; Figure 1F3-C). OCT showed a wrinkled, elevated macular area and the fovea centralis disappeared of the left eye. Macular OCT could not be performed in the right eye due to retinal detachment (Figure 1F3-C). The results of binocular color ultrasonography showed both eyes to have short axes, and the right eye to have retinal detachment coupled with choroidal detachment. ERG findings suggested a severe decrease in b-wave amplitude in the scotopic 0.01, scotopic 3.0, and photopic 3.0 responses in the right eye, and a severe decrease in flicker response and amacrine cells function. In the left eye, the b-wave amplitude was mildly decreased in the scotopic 0.01 and scotopic 3.0, the b-wave amplitude was within the normal range in the photopic 3.0 response, the response of flicker was severely decreased, and the amacrine cells function was basically normal (Table 2). As confirmed by the whole exome sequencing and Sanger sequencing, the patient carried a homozygous missense variant c.1486G>A (p.Glu496Lys) in the *MFRP* gene, and both the phenotypically normal father and mother carried the same heterozygous variant, suggesting co-segregation of genotypes and clinical phenotypes (Figure 1F3-B; Table 3) (PP1_supporting), which is consistent with an autosomal recessive pattern of inheritance. Such variant was not detected in the East Asian population database (ExAC_ EAS), gnomAD database (PM2_Moderate). A variety of bioinformatics computing software indicated the deleterious impact of this variant (PP3_supporting). Proteomic conservation analysis revealed that the amino acid at site 496 was highly conserved across multiple species (Figure 1D). The protein structure suggested that the mutation will result in protein structure and function. (Figure 1E; Figure 3). According to the ACMG guidelines, the c.1486G>A (p.Glu496Lys) missense variant was classified as a likely pathogenic (PP1+PM2+PP3+PP4+PP5).

### 3.4 Pedigree 4

**FIGURE 4:**
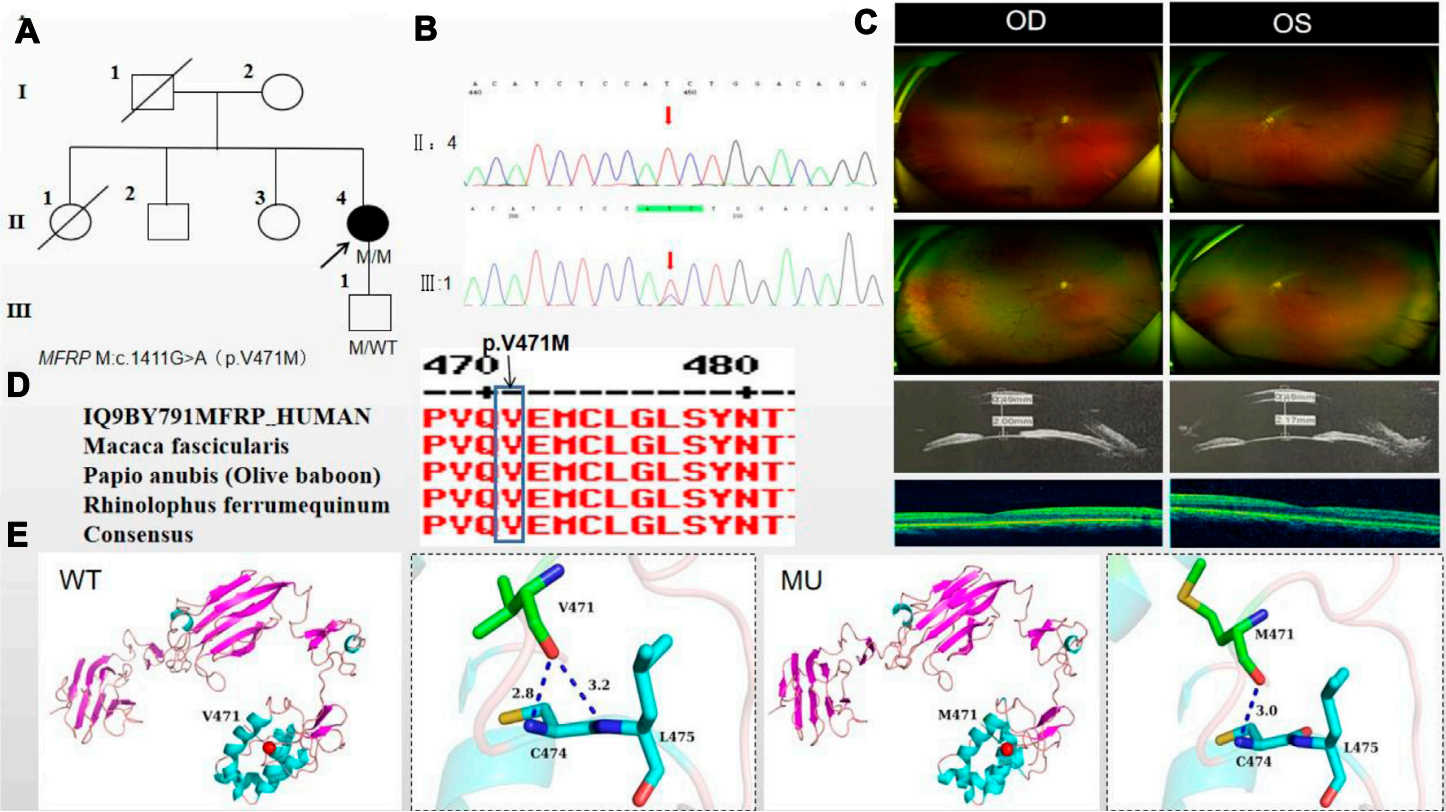
Sequence analysis and clinical examination of Family 4. **(A)** Pedigree of Family 4. The filled black symbols represent the affected members, and the arrow denotes the proband. **(B)** Sequence chromatograms of identified mutations. **(C)** The fundus of both eyes: The fundus examination revealed that small optic discs with clear borders in both eyes, C/D ratio was 0.8, with markedly dilated retinal veins in the right eye, but mildly dilated retinal veins in the left. A large number of scattered hemorrhagic spots were found on the retinal surface of the right eye at follow-up for up to 2 months. UBM showed the anterior chamber depth of right eye and left eye were 2.00 mm and 2.17 mm, respectively. OCT showed the macular area was wrinkled and mildly edematous, and the fovea centralis disappeared in the right eye and was visible in the left eye. **(D)** The homology of amino acid sequences between human MFRP and other species. The amino acid at position 471 is highly conserved among species. The mutated residue 471 is boxed and indicated. **(E)** The protein structure suggested that the p. Val471Met mutation resulted in the substitution of the nonpolar uncharged valine at site 471 of the MFRP protein for a nonpolar uncharged methionine.

The proband of family 4, a female, age of 41, complained of reduced vision accompanied by occlusion for 3 months. She denied consanguineous marriage (Figure 4A). BCVA was 0.04 in the right eye and 0.15 in the left eye. The axial length of right eye and left eye were 18.25 mm and 18.30 mm, respectively. The anterior chamber depth of right eye and left eye were 2.00 mm and 2.17 mm, respectively (Figure 4C). The intraocular pressure was 27 mmHg in the right eye and 23 mmHg in the left eye (Table 1), and she had not received any treatment before joinning the study. Static gonioscopy showed closure of the anterior chamber angle in all four quadrants, all of which were narrow IV. Fundus examination revealed that small optic discs with clear borders in both eyes, C/D ratio was 0.8, with markedly dilated retinal veins in the right eye, but mildly dilated retinal veins in the left. A large number of scattered hemorrhagic spots were found on the retinal surface of the right eye at follow-up for up to 2 months. OCT showed the macular area was wrinkled and mildly edematous, and the fovea centralis disappeared in the right eye and was visible in the left eye (Figure 3C). ERG findings suggested b-wave amplitude higher than normal in the scotopic 0.01, scotopic 3.0 and photopic 3.0, responses of flicker were also higher than normal range in the left eye; b-wave amplitude and response of flicker in the normal range in the scotopic 0.01 in the right eye, the b-wave amplitude was higher than normal in the scotopic 3.0 and photopic vision 3.0, the amakrine cells function was basically normal (Table 2). As confirmed by the whole exome sequencing and Sanger sequencing, the patient carried a homozygous missense variant c.1411G>A (p.Val471Met)in the *MFRP* gene, and the phenotypically normal son carried the same heterozygous variant, suggesting co-segregation of genotypes and clinical phenotypes (Figure 3B; Table 3), which is consistent with an autosomal recessive pattern of inheritance (PP1_Supporting). Such variant was not detected in the East Asian population database (ExAC_ EAS), gnomAD database (PM2_Moderate). A variety of bioinformatics computing software indicated the deleterious impact of this variant (PP3_supporting). Proteomic conservation analysis revealed that the amino acid at site 471 was highly conserved across multiple species (Figure 4D), indicating that variants at such site are likely to have an impact on the structure and function of the MFRP protein. In the wild-type MFRP protein, site 471 was a nonpolar uncharged valine, and the O atom of its amino acid backbone formed a hydrogen bond with the O atom of the uncharged polar cysteine at site 474 and the uncharged nonpolar amino acid at site 475, and the p. Val471Met mutation resulted in the substitution of the nonpolar uncharged valine at site 471 of the MFRP protein for a nonpolar uncharged methionine. Such mutation led to the disappearance of the hydrogen bond formed between the O atom of the backbone and the uncharged nonpolar leucine at site 475, resulting in alterations in the structure and function of the protein (Figure 4E). According to the ACMG guidelines, the c.1411G>A (p.Val471Met)missense variant was rated as PP4 due to the phenotype of the missense variant carrier was highly consistent with nanophthalmos, and genetic testing report from credible sources suggested that the variant was pathogenic as PP5. Therefore, the c.1411G>A (p.Val471Met) missense variant was classified as a likely pathogenic according to the ACMG guidelines (PP1+PM2+PP3+PP4+PP5).

### 3.5 Pedigree 5

**FIGURE 5:**
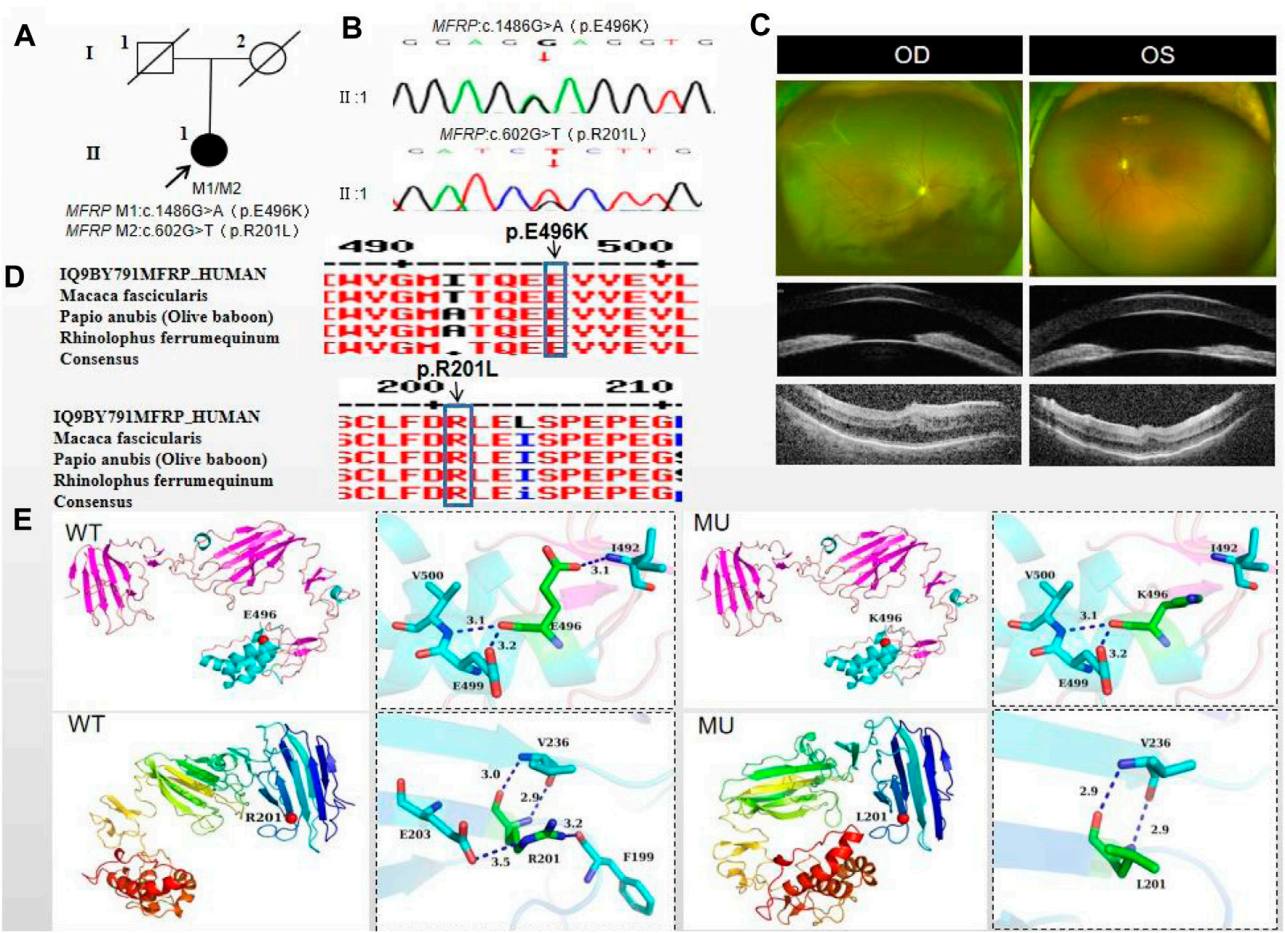
Sequence analysis and clinical examination of Family 5. **(A)** Pedigree of Family 5. The filled black symbols represent the affected members, and the arrow denotes the proband. **(B)** Sequence chromatograms of identified mutations. **(C)** The fundus of both eyes: The fundus examination revealed that small optic discs with clear borders in both eyes, C/D ratio was 0.8, with markedly dilated retinal veins in the right eye, but mildly dilated retinal veins in the left. A large number of scattered hemorrhagic spots were found on the retinal surface of the right eye at follow-up for up to 2 months. UBM showed the anterior chamber depth of right eye and left eye were 2.00 mm and 2.17 mm, respectively. OCT showed the macular area was wrinkled and mildly edematous, and the fovea centralis disappeared in the right eye and was visible in the left eye. **(D)** The homology of amino acid sequences between human *MFRP* and other species. The amino acid at position 471 is highly conserved among species. The mutated residue 471 is boxed and indicated. **(E)** The protein structure suggested that the p. Val471Met mutation resulted in the substitution of the nonpolar uncharged valine at site 471 of the MFRP protein for a nonpolar uncharged methionine.

The proband of family 5, a female, age of 43, complained of reduced vision for 6 months. She denied a family history of hereditary disease and consanguineous marriage history (Figure 5A). BCVA was 0.15 in the right eye and 0.2 in the left eye. The axial length of right eye and left eye were 15.28 mm and 15.03 mm, respectively. The anterior chamber depth of right eye and left eye were 1.91 mm and 2.00 mm, respectively (Figure 5C). The intraocular pressure was 30 mmHg in the right eye and 28 mmHg in the left eye (Table 1), and she had not received any treatment before joinning the study. Static gonioscopy showed closure of the anterior chamber angle in all four quadrants, all of which were narrow IV. Fundus examination revealed that small optic discs with clear borders in both eyes, C/D ratio was 0.8, with mildly dilated retinal veins. Macular spectral-domain optical coherence tomography of both eyes shows a small dome-shaped papillomacular fold and nomacular edema (Figure 5C). ERG findings suggested that the b-wave amplitude in the scotopic 0.01 and 3.0 responses was within the normal range, the flicker response was severely decreased, and amakrine cells functioned normally. As confirmed by whole exome sequencing and Sanger sequencing, a novel compound heterozygous missense variants c.1486G>A (p.Glu496Lys) and c.602G>T (p.Arg201Leu) were detected on the *MFRP* gene of the proband (Figure 5B; Table 3) (PP1_supporting). The parents of the proband were deceased, and the genetic origin was unclear. However, it can be considered an autosomal recessive pattern of inheritance according to genetic test results and clinical phenotype. Such variant was not detected in the East Asian population database (ExAC_ EAS) and the gnomAD database (PM2_Moderate), and a variety of bioinformatics computing software indicated the deleterious impact of this variant (PP3_supporting). Proteomic conservation analysis revealed that the amino acid at site 496 and 201 were highly conserved across multiple species (Figure 5D). The protein structure suggested that the 201th position of wild-type MFRP protein is arginine with positive polarity, and the N atom in the main chain forms hydrogen bonds with the uncharged nonpolar valine O atom in the 236th position, and the O atom in the main chain forms hydrogen bonds with the valine N atom, and the side chains of amino acids form hydrogen bonds with the F199 position and E203. The mutation of p. Arg20Leu leads to the substitution of arginine at position 201 by nonpolar leucine, and the N atom of leucine main chain forms hydrogen bonds with uncharged nonpolar valine O atom at position 236, and the O atom of main chain forms hydrogen bonds with valine N atom, and the nonpolar functional groups of side chains do not interact with the surrounding amino acids. The changes of amino acid interaction and amino acid properties lead to the changes of protein structure and function after mutation. The missense variant c.1486G>A (p.Glu496Lys) resulted in the substitution of the polar negatively charged glutamic acid for a polar positively charged lysine at site 496 of the MFRP protein, which resulted in alterations in protein structure and function (Figure 5E). Therefore, the c.1486G>A (p.Glu496Lys) and c.602G>T (p.Arg201Leu) missense variant was classified as a likely pathogenic according to the ACMG guidelines (PM2+ PP1+PP3+PP4+PP5).

## 4 Discussion

Nanophthalmos is characterized by reduced age-adapted axial length of the eye leading to high hyperopia ranging from +8.00 to +25.00 dioptres (D) (Relhan et al., 2016). It can be isolated, or accompanied by other ocular anomalies (Ghose et al., 1985) or systemic abnormalities (Dos Santos Martins et al., 2023). The main manifestations of nanophthalmos include thickening and hardening sclera, reduced anterior chamber depth, steep or irregular corneas, disproportionally large lens, foveal hypoplasia, loss of foveal avascular zone, papillomacular puckers, foveoschisis, optic disc drusen and retinal dystrophy.

Nanophthalmos can be regarded as a class of eye diseases with extreme developmental abnormalities of the axial length and chamber angle. The study of nanophthalmos in Chinese patients has great significance for understanding the occurrence and development of axial length, shallow anterior chamber, narrow chamber angle and angle-closure glaucoma in Asian population. In addition, the study of the regulation mechanism of ocular axial length may also open new ideas for the study of myopia, because the mechanism responsible for the “short axial length " may colocalizes with the pathway involved in the long axial length (myopia). In addition to these clinical features in the anterior segment of the nanophthalmos, the choroid and sclera are thicker in patients than in healthy eyes (Rajendrababu et al., 2022; Fernández-Vigo et al., 2023). Therefore, the underlying mechanism of nanophthalmos can be used to study the anterior and posterior segments.

The membrane frizzled-related protein (*MFRP*) gene is located on chromosome 11q23 and encodes a glycosylated transmembrane protein that has a frizzled-related cysteine-rich domain (Kautzmann et al., 2020). The frizzled protein is a receptor involved in the regulation of growth, development and cell polarity through the Wnt signaling pathway (Bhanot et al., 1996; Katoh, 2001). The protein structure consists of two cubilin-related domains (CUB), two low-density lipoprotein receptor class A domains (LDLa), and a cysteine rich domain (CRD) (Won et al., 2008). *MFRP* is mainly expressed in the ciliary body and retinal pigment epithelium (Sundin et al., 2008). It plays an important role in ocular growth during childhood, as well as in the maintenance of RPE photoreceptor function (Mameesh et al., 2017). The *MFRP* gene was initially associated only with high hyperopia and reduced axial length, but not with retinal degeneration. However, since 2006, a series of cases have been reported *MFRP* mutations are associated with posterior nanophthalmos, RP, macular abnormalities, and optic disc drusen (Mameesh et al., 2017).

In this study, the proband of family two from a families with consanguineous marriage that carried the homozygous nonsense variant c.271C>T (p.Gln91Ter), such variant caused truncations of the MFRP protein. As reported previously, fundus examination of patients associated with *MFRP* syndrome usually showed multiple irregular round yellowish-white spots located in the mid-periphery of the retina, with or without osteochromic pigmentation, and some patients may be complicated with optic disc drusen (Crespí et al., 2008). The fundus of the proband in this study had pigmentation of peripheral retina 360° from the equator to the serrated rim showing a clear demarcation from the normal retina in the form of strips, which was completely different from the fundus features previously reported in the literature. It is noteworthy that c.271C>T (p.Gln91Ter) detected in this study was previously reported as a compound heterozygous variant with c.498dupC (p.Asn167fs) in a German microphthalmic family (Crespí et al., 2008) and it reported as pathogenic. The two patients in this family had an ocular axis length of 15 mm, macular puckers, optic disc drusen and essentially normal autofluorescence, whereas the macular OCT in our study showed alterations similar to congenitalretinoschisis and was not complicated with optic disc drusen, suggesting that, like other genetic disorders, *MFRP*-associated microphthalmos was characterized by clinical and genetic heterogeneities.

In this study, both family one and family three carried the same c.1486G>A (p.Glu496Lys) missense variant of the *MFRP* gene. The two families are from different regions of Ningxia, and there is no kinship between the two families. The clinical phenotypes of two probands were distinctly different. The severity of fundus lesions in family three patients was significantly higher than family 1. Notably, as early as 2009, studies have shown that certain clinical phenotypes of nanophthalmos caused by *MFRP* mutations are correlated with age (Zenteno et al., 2009). Since then, a Dutch study in 2014 also pointed out that all patients with autosomal recessive inherited *MFRP* mutations exhibit high hyperopia, but the effect of the mutation on retinal photoreceptor function varies from person to person, and these affected patients usually develop clinical signs of photoreceptor dysfunction or retinal dystrophy later in life (Zenteno et al., 2009). These data suggest that *MFRP* mutations result in the fundus phenotype characteristic of “typical” retinitis pigmentosa, with retinal pigmentary changes occurring as fine mottling or granularity with surrounding areas of atrophy in the early stages followed by the typical “bone spicule” pattern of hyperpigmentation in the midperipheral retina, which suggested that the extent of retinal degeneration is age-dependent. In addition, nanophthalmos is characterized by spontaneous uveal effusion syndrome with age, which is closely related to scleral thickening with age and obstruction of vortex venous return. In this study, the proband in family 1, 7 years old, had mild fundus phenotype, whereas the proband in family 3, 17 years old, had a severe phenotype, which could not exclude an age-related association. The reason for this clinical heterogeneity may be the result of a combination of the mutational profile of the *MFRP* gene and other genetic or environmental factors to be determined. Of course, further subsequent studies in other subjects of different ages are needed to validate our view.

Family four proband carried the *MFRP* homozygous missense mutation c.1411G>A (p.Val471Met). This variant has been reported as a compound heterozygous variant with c. 661C>T (p.Pro221ser) in the Chinese population, but the authors failed to provide a detailed description of the clinical phenotypes (Guo et al., 2019a), whereas the patients carrying this variant locus in this study had high intraocular pressure in both eyes, shallow anterior chamber, and closure of the anterior chamber angle in all quadrants. Fundus examination revealed that small optic discs with clear borders in both eyes, C/D ratio was 0.8, which was considered chronic angle-closure glaucoma in both eyes. In addition, the patient was complicated with retinal vein obstruction in the right eye during follow-up, and a large number of scattered hemorrhagic spots appeared on the retinal surface. The first case of nanophthalmos complicated by retinal vein obstruction was reported in 2015 by American scholars. The patient was not suffering from systemic diseases at the time. The fundus examination results showed no obvious abnormalities in the retina except for the macular puckers in the right eye, while the fundus examination of the left eye showed flame-shaped hemorrhages in the superior half of the retina with macular edema and optic disk congestion. Unfortunately, the patient was not genetically tested (Albar et al., 2015). In this study, a large number of scattered punctate hemorrhages were observed on the retinal surface of family four proband, accompanied by mild macular edema, but no congestion and edema were observed in the optic disc. This seems to provide a possible influencing factor for retinal vein occlusion, but whether nanophthalmos is a risk factor for retinal vein occlusion is still controversial and needs further study.

Family five probands carried compound heterozygous missense variants c.1486G>A (p.Glu496Lys) and c.602G>T (p.Arg201Leu), which had the same mutation c.1486G>A (p.Glu496Lys) as family one and family 3. The probands presented clinically with binocular glaucoma, which is very different from the clinical phenotypes of family one and family 3, potentially indicating a connection to genetic variability. In addition, we also found that the occurrence of glaucoma was age-dependent in nanophthalmos. As several studies have shown (Guo et al., 2019b; Khan, 2020) that glaucoma was not observed in young patients with nanophthalmos, whereas there are also studies showed glaucomatous manifestations in middle-aged and elderly patients (ernández-Vigo et al., 2023),. In this study, the probands of family 1, family two and family three were young and had not yet shown glaucoma, whereas the middle-aged patient of family four and family five were considered to have chronic angle-closure glaucoma, which was also consistent with previous studies and was considered to be correlated with the increase in age and lens thickness. Of course, further studies in patients of different ages are needed to clarify the point.

As a final note, both three families carried the c.1486G>A (p.Glu496Lys) missense variant of the *MFRP* gene, it appears in 3/5 cases in this study. We concluded that it may be related to the small sample size of this group and the fact that two of the families were from the Hui nationality. Of course, we could not rule out c.1486G>A may occur more frequently in nanophthalmos patients in the Chinese population, which should be validated by a larger sample size investigation.

Significant progress has been made in the diagnosis and treatment of nanophthalmos in recent years with the continuous development of imaging and surgery. In addition, advances in genetics research can help us better understand the pathogenesis of nanophthalmos and offer the possibility of gene therapy in the future. Genetic diagnoses will facilitate genetic counseling for forms of this condition and may help to decrease amblyopia from uncorrected hyperopia, prevent vision loss from complications, and improve monitoring to minimize glaucoma and retinal complications from nanophthalmos.

## 5 Conclusion

Nanophthalmos is a complex disease with high genetic and phenotypic heterogeneity. Its clinical complications are serious enough to warrant early diagnosis and prompt genetic counseling. Here, in this paper, we identified four homozygous variants and one complex heterozygous variants in the *MFRP* gene in five Chinese families and concluded that the *MFRP*-nanophthalmos encompass a features of distinct phenotypic diversity. Our findings extend the phenotype associated with *MFRP* variants and is helpfull for ophthalmologists in early diagnosis and making effective treatment and rehabilitation strategies. Noteworthly, although *in silico* analyses of the effects of the identified mutations suggest they likely alter protein structure, whether they alter protein function would need to be proven experimentally, and the related studies for deeply and thoroughly understand this problem should be performed in future.

## Data Availability

The datasets presented in this study can be found in online repositories. The names of the repository/repositories and accession number(s) can be found in the article/Supplementary Material.

## Abbreviations

ACMG, American College of Medical Genetics; P, probably damaging; D, damaging; T, tolerated; ND, not done; N, neutral; PVS1, very strong null variant; PM2, absent from controls (or at extremely low frequency if recessive) in Exome Sequencing Project, 1000 Genomes Project, or Exome Aggregation Consortium; PP1, Congregation with disease in multiple affected family members in a gene definitively known to cause the disease. May be used as stronger evidence with increasing segregation data; PP3, multiple lines of computational evidence support a deleterious effect on gene or gene product; PP4, patient’s phenotype or family history is highly specific for a disease with a single genetic etiology; PP5, peputable source recently report variant as pathogenic.
